# Investigation of Ground-Level Ozone and High-Pollution Episodes in a Megacity of Eastern China

**DOI:** 10.1371/journal.pone.0131878

**Published:** 2015-06-29

**Authors:** Heng Zhao, Shanshan Wang, Wenxin Wang, Rui Liu, Bin Zhou

**Affiliations:** 1 Shanghai Key Laboratory of Atmospheric Particle Pollution and Prevention (LAP^3^), Department of Environmental Science & Engineering, Fudan University, Shanghai 200433, China; 2 School of Environment and Architecture, University of Shanghai for Science and Technology, Shanghai 200093, China; 3 Fudan Tyndall Centre, Fudan University, Shanghai 200433, China; Nanjing University, CHINA

## Abstract

Differential Optical Absorption Spectroscopy (DOAS) was used for the long-term observation of ground-level ozone (O_3_) from March 2010 to March 2013 over Shanghai, China. The 1-hour average concentration of O_3_ was 27.2 ± 17.0 ppbv. O_3_ level increased during spring, reached the peak in late spring and early summer, and then decreased in autumn and finally dropped to the bottom in winter. The highest monthly average O3 concentration in June (41.1 ppbv) was nearly three times as high as the lowest level recorded in December (15.2 ppbv). In terms of pollution episodes, 56 hourly samples (on 14 separate days) in 2010 exceeded the 1-hour ozone limit of 200 μg/m^3^ specified by the Grade II of the Chinese Ambient Air Quality Standards (CAAQS, revised GB 3095-2012). Utilizing the Hybrid Single Particle Lagrangian Integrated Trajectory (HYSPLIT) model, the primary contribution to high ozone days (HODs) was identified as the regional transportation of volatile organic compounds (VOC) and high concentrations of O_3_ from the chemical industrial zone in the Jinshan district of Shanghai. HODs showed higher concentrations of HONO and NO_2_ than non-episode conditions, implying that HONO at high concentration during HODs was capable of increasing the O_3_ concentration. The photolysis rate of HONO was estimated, suggesting that the larger number of OH radicals resulting from high concentrations of HONO have a considerable impact on ozone concentrations.

## Introduction

Ground-level ozone (O_3_) is regarded as one of the most significant atmospheric photochemical products. Due to the increasing motorized traffic, industrial and agricultural activities, tropospheric ozone concentrations increased substantially in recent decades [1, 2]. Dense ozone near ground level adversely affects human health, ecological system, and cultural heritage buildings [3–5]. Studies on ground-level ozone have contributed greatly to improving urban air quality and understanding the impact of tropospheric ozone on the environment. The factors favoring concentrations of ground-level ozone include high ambient temperature, intense photochemical reactions between NO_X_ and VOCs, and thin boundary layer. In addition, surface ozone concentration can be strongly affected by meteorological conditions such as solar radiance, relative humidity, and wind speed [6].

Shanghai is one of the largest megacities in eastern China, experiencing rapid urbanization and industrialization. The increases of population, industrial activity, and automobiles are conducive to significantly increased emissions of VOCs and NO_x_. The atmospheric abundance of these compounds subsequently influence O_3_ production. High ozone episodes and related photochemistry in Shanghai have been reported in previous papers, which were usually occurred in spring and summer. Ran [7] found that spring was the most productive season for ozone, with the highest daily maximum (128 ppbv) in May 2007 in Shanghai. High ozone periods with daily maximum ozone exceeding 102 ppbv were observed typically lasting for 3~5 days at a rural site of Shanghai in August 2010 [8]. The ozone weekly cycle that higher concentrations at weekend and lower during weekdays in Shanghai urban site is caused by different NO_2_/NO ratio and the rate of ozone production is a function of atmospheric VOCs/NO_x_ ratio [9]. Besides, previous studies showed that the ozone formation in Shanghai was limited by the VOCs concentrations [7, 10, 11]. Moreover, the complex monsoon in Shanghai significantly affects atmospheric pollution via air mass transport. Wang [12] found that the summer monsoon introduced oceanic air with lower ozone concentration to the region, and caused lower ozone mixing ratios in summer, such that peak ozone concentrations occurred during late spring at sites in the Yangtze River Delta. Therefore, as the research hotspot in atmospheric chemistry, O_3_ production and destruction in Shanghai still deserve to be better understood, which is a critical prerequisite for the development of effective O_3_ control strategies.

Differential Optical Absorption Spectroscopy (DOAS) is a well-established technique and has been used to measure trace gases such as O_3_, NO_2_, SO_2_, HONO, HCHO, aromatics and halogen oxides worldwide [13–15]. Several previous studies used DOAS to measure tropospheric air pollution [16–21]. Premuda [22] analyzed the vertical structure of O_3_ and NO_2_ concentrations measured by DOAS on the Castel Porziano Presidential Estate pine forest near a metropolitan area. Observation for SO_2_, NO_2,_ and O_3_ concentrations by the DOAS technique in an urban semi-industrial area of Athens, Greece, provided the evidence of higher ozone concentrations during weekends despite lower concentrations of ozone precursors [23]. In Shanghai, the active DOAS technique was applied to measure atmospheric SO_2_, NO_2_, O_3_, HONO, HCHO and NO_3_ in previous researches [24–27].

In this study, measurements of ground-level O_3_ were performed by the DOAS technique from March 2010 to March 2013 in Shanghai, China. These long-term data series were used for preliminary assessment of O_3_ temporal characteristics, e.g. seasonal and diurnal patterns of O_3_ concentration in Shanghai. The relationships between ozone and its photochemical precursors, such as NO_2_ and HONO, are discussed with regard to ozone formation mechanism. For more detailed insight into patterns of ozone pollution, HODs (high ozone days) were analyzed with HONO trend (a significant precursor of ozone) and backward trajectory. Additionally, the production of hydroxyl radicals from photolysis of HONO was estimated, and air mass back-trajectories and meteorological parameters were used to further analysis of the contributory factors to extreme O_3_ concentration.

## Data and Methods

### Measurement site and experimental setup

The measuring principle of a DOAS system is based on the fact that all trace gases absorb electromagnetic radiation in some part of the spectrum. The result of the absorption measurement is evaluated in terms of the correlation between the gas concentration and the amount of light absorbed, following the Lambert-Beer law:

$$I\left(\lambda \right)={Ι}_{0}\left(\lambda \right)\times e^{-\sigma \left(\lambda \right)cL}$$ (1)

Here, I_0_(*λ*) is the reference spectrum; I(*λ*) is the spectrum at a distance L through the atmosphere; *σ*(*λ*) is the absorption cross-section at wavelength *λ*; c is the concentration of gas; L is the light path length [15]. There were no specific permissions required for this experiment location, and the experiment study did not involve endangered or protected species.

**Fig 1:**
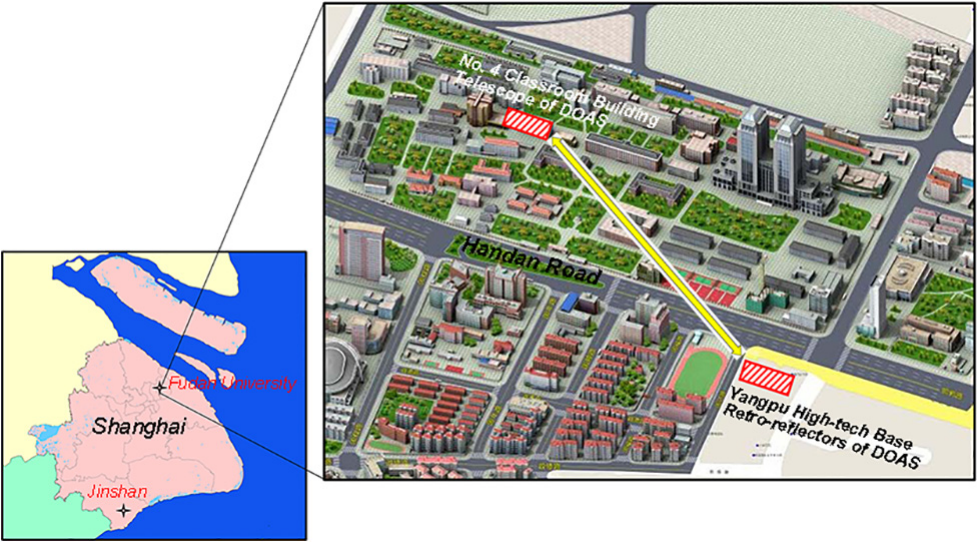
Overall view of the measurement site and light path of the DOAS system.

The DOAS system designed and assembled by the authors consists of a telescope of diameter 210 mm as transmitter and receiver, a 150 W xenon lamp as light source, and a spectrograph (B&W TEK Inc. BRU741E-1024) with a spectral range of 200–450 nm and spectral resolution (FWHM) of 0.75nm. It was operated at the center of Fudan University (FDU: 31.3°N, 121.5°E), Shanghai, China. The transmitting/receiving telescope of the DOAS system was located on the roof of the No. 4 Classroom Building at a height of 20 m above the ground. The retro-reflectors were mounted at a height of approximately 44 m at the Yangpu High-tech Base building, which is located 0.68 km southeast of the classroom building. The measurement site and light path of the active DOAS system are shown in Fig 1. For the instrument maintenance, the surface of retro-reflectors and the window of receiver side were cleaned routinely every two weeks to scavenge the deposited dust and dirty.

The DOAS light beam travels above the campus from northwest to southeast crossing Handan Road, which is a trunk road with heavy traffic and an expressway tunnel beneath, running 300 m south of the No. 4 Classroom building. Moreover, there are several branch roads around the campus. Vehicular emissions are responsible for the major source of NO_x_ near the measurement site. A large petrochemical complex and fine chemistry are located in the Jinshan district, approximately 60 km southwest of the monitoring site (see Fig 1).

### Data analysis

**Fig 2:**
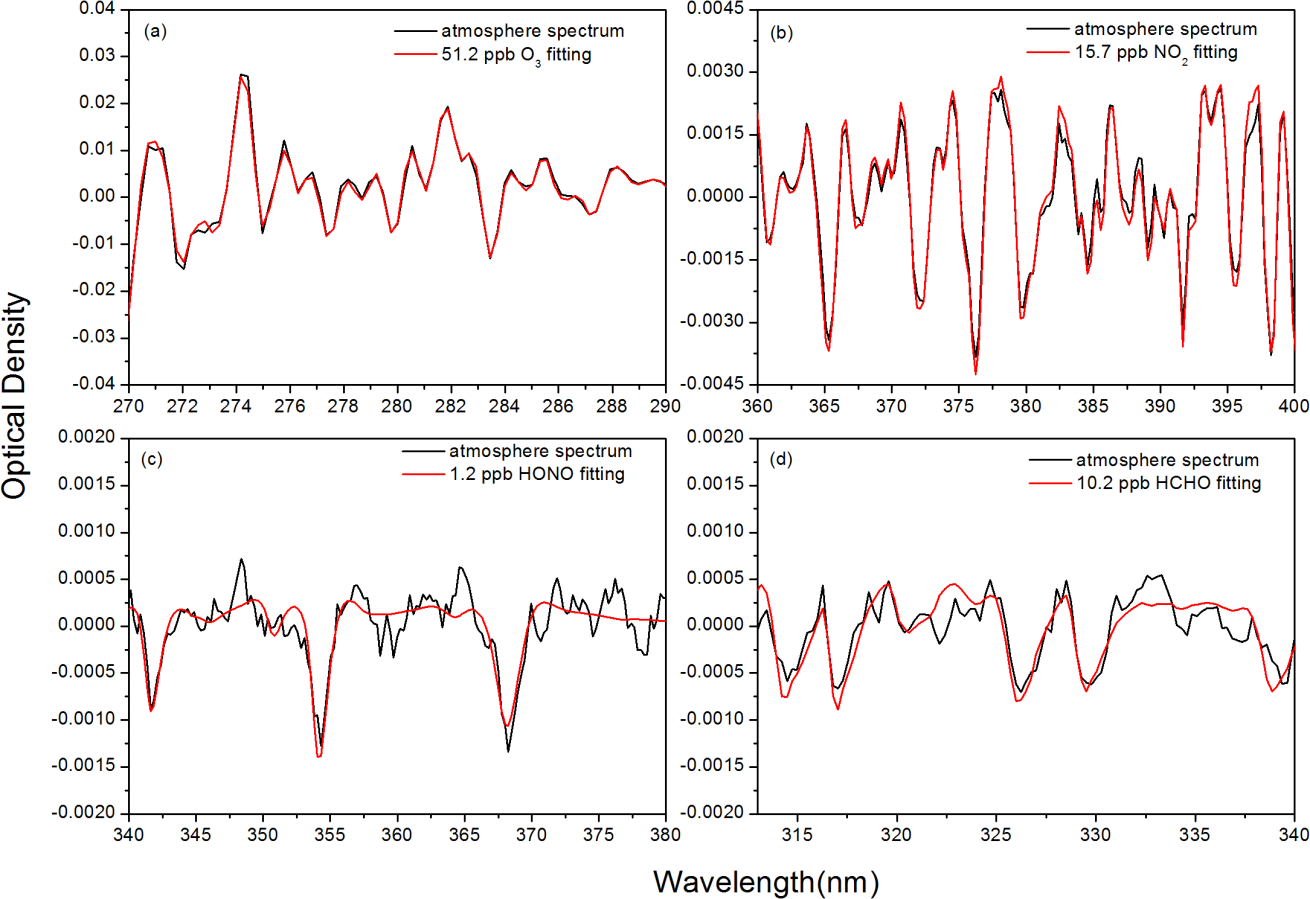
Examples of DOAS fitting for O_3_, NO_2_, HONO and HCHO.

The spectra were analyzed by DOASIS software (Institute of Environmental Physics, IUP, Heidelberg University, Germany). O_3_ is measured within the range 270–290 nm; NO_2_ at 360–400 nm; HCHO at 313–340 nm and HONO at 340–380 nm with a 3-min temporal resolution. High-resolution absorption cross-sections of O_3_ [28], NO_2_ [29], HONO [30] and HCHO [31] were used in the spectra fitting (details in Table 1). Fig 2 shows the examples of DOAS fit for O_3_, NO_2_, HONO and HCHO. Ground-based active DOAS measurements for O_3_, NO_2_, HONO and HONO were conducted from March 2010 to March 2013. A total of 191 695 spectra were analyzed, excluding some gaps due to maintenance of the instruments, inclement weather (e.g., fog and heavy rain), and shifts in the light path.

**Table 1:** Overview of DOAS analysis settings for the measured species.

Species	Cross Sections	Wavelength (nm)	Polynomial (Order)	Detection Limits^1^ (ppbv)
O_3_	O_3_, SO_2_, O_2_, HCHO, CH_3_CHO	270–290	3rd	3.0
NO_2_	NO_2_, HONO, HCHO	360–400	3rd	2.0
HONO	HONO, NO_2_, HCHO, SO_2_	340–380	3rd	0.2
HCHO	HCHO, SO_2_, NO_2_,O_3_	313–340	3rd	1.0

^1^ according to instruments noise in a light path of 1.36 km and 3 min integration time.

Meteorological data with a 5-min temporal resolution were collected at Pudong meteorological site (31.1°N, 121.5°E) in Shanghai, located approximately 10 km from the FDU site. To address the different temporal resolution between DOAS measurements and meteorological data, the meteorological data were averaged for hourly means to be discussed.

The 24-h air mass back-trajectories were calculated using the Hybrid Single Particle Lagrangian Integrated Trajectory (HYSPLIT) model (Version 4: Air Resources Laboratory, NOAA: National Oceanic and Atmospheric Administration, USA), which can identify the origins and transport of air masses arriving at the FDU measurement site. The meteorological data used in HYSPLIT model are the Global Data Assimilation System (GDAS) datasets with a spatial resolution of 1° × 1° and 24 vertical levels.

## Results and Discussion

### O_3_ concentration

**Fig 3:**
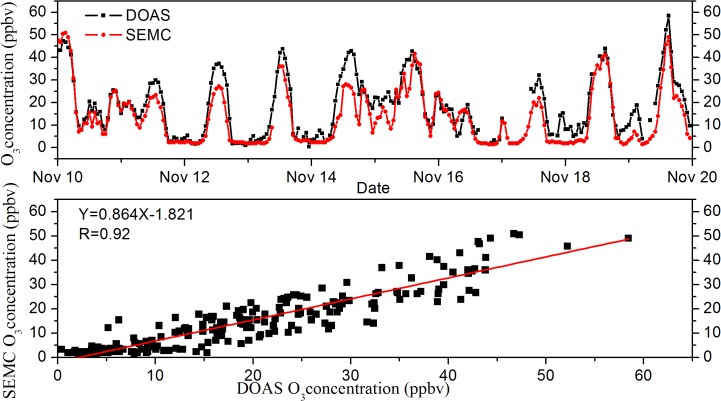
Comparison of O_3_ data measured by DOAS and SEMC temporal variation from November 10 to 19, 2012.

**Fig 4:**
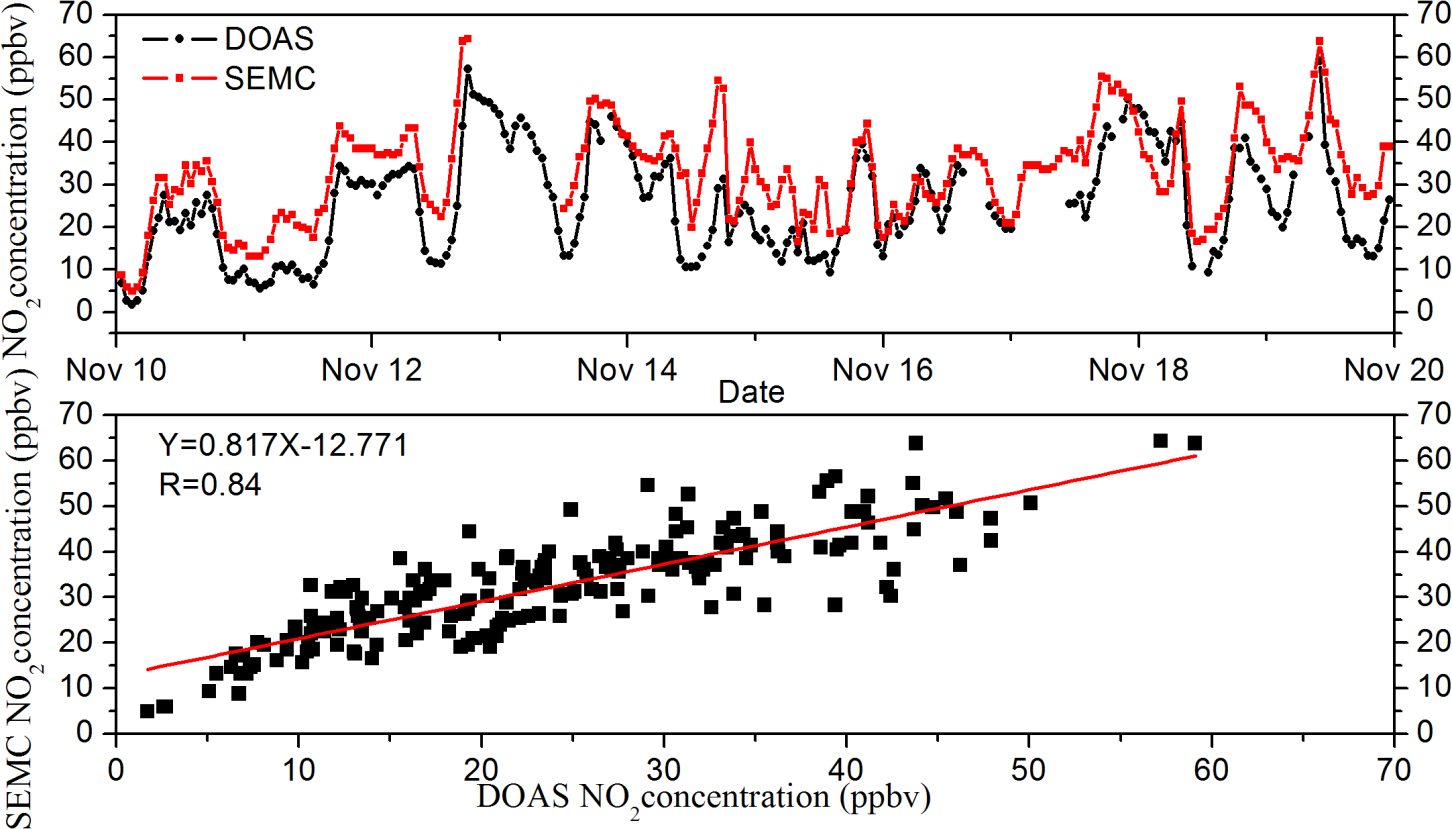
Relationship between concentrations measured via DOAS and SEMC.

To demonstrate the reliability of DOAS observation, O_3_ concentrations were compared with in-situ measurement by SEMC (Shanghai Environmental Monitoring Center, data from www.semc.gv.cn), located about 3 km away from FDU site, from Nov.10 to Nov.19, 2012. As shown in Fig 3 and Fig 4, the DOAS technique show a good performance in O_3_ and NO_2_ measurements compared to the SEMC data. The concentrations of O_3_ and NO_2_ measured by these two techniques generally coincided with each other, showing correlation coefficients R of 0.92 and 0.84, respectively. This reasonable difference between DOAS and in-situ measurement was probably due to the individual technique principle and distinct measurement site environment.

**Fig 5:**
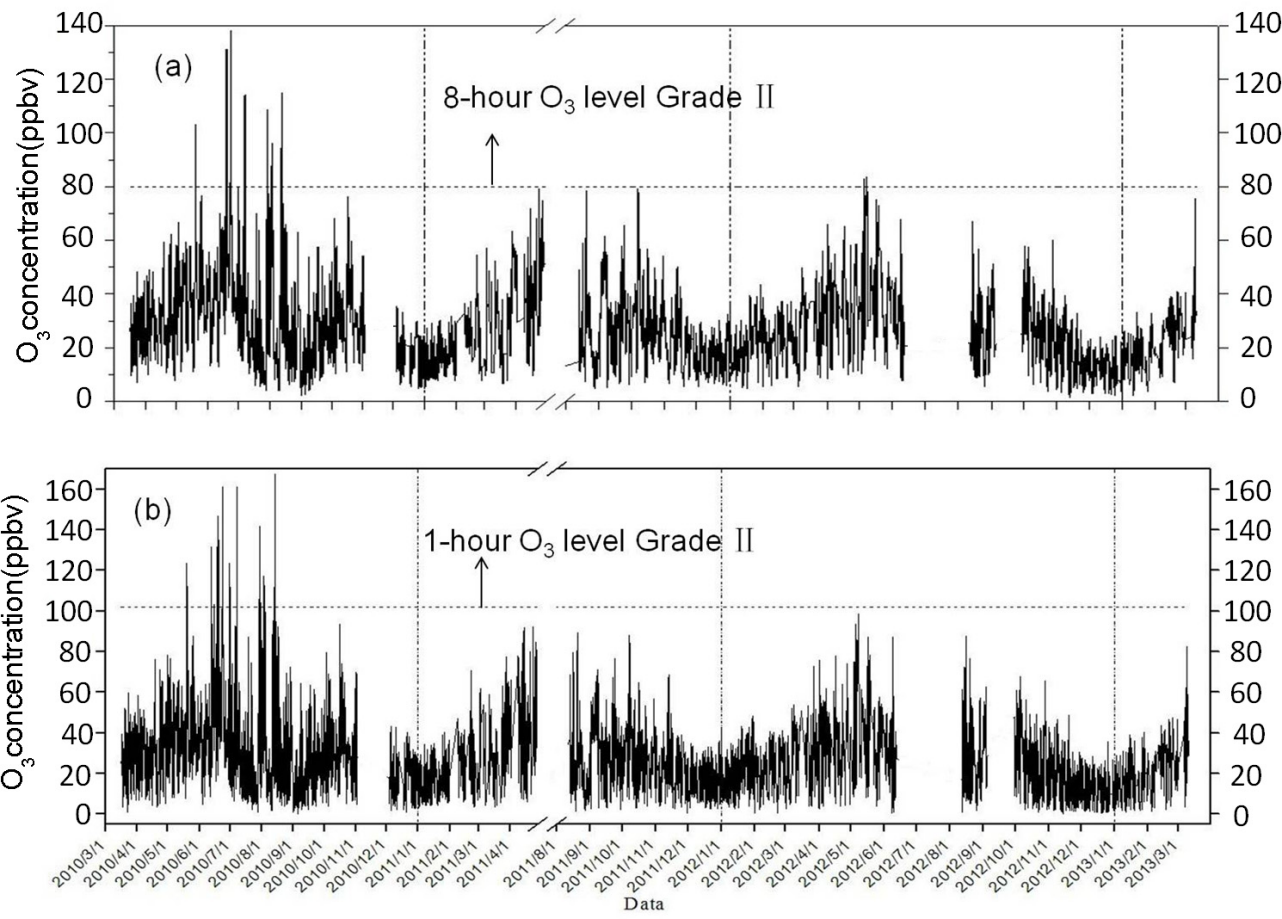
Time series of 8-h and 1-h average O_3_ levels.

Time series for 8-hour and 1-hour average O_3_ concentrations from March 2010 to March 2013 are displayed in Fig 5. The 8-hour moving average O_3_ concentration ranged from 8.5 ppbv to 138.0 ppbv with an average of 27.2 ppbv and standard deviation of 14.8 ppbv. The maximum 8-h ozone concentration occurred from 11:00 to 19:00 on June 13, 2010, and the 8-hour moving average O_3_ concentrations exceeded Grade II (160 μg/m^3^, approximately 81 ppbv) of the Chinese Ambient Air Quality Standards (CAAQS, revised GB 3095–2012) among 14 days. The 1-h average O_3_ concentration ranged from <1 ppbv to 167.3 ppbv with an average of 27.2 ppbv and standard deviation of 17.0 ppbv. The highest hourly average (167.3 ppbv) occurred at 15:00 on August 13, 2010, and 56 hourly O_3_ concentrations among 14 different days, shown in Fig 5(B), exceeded the Grade II threshold for hourly data (200 μg/m^3^, approximately 102 ppbv). The average O_3_ level in summer was significantly higher than that in winter.

**Fig 6:**
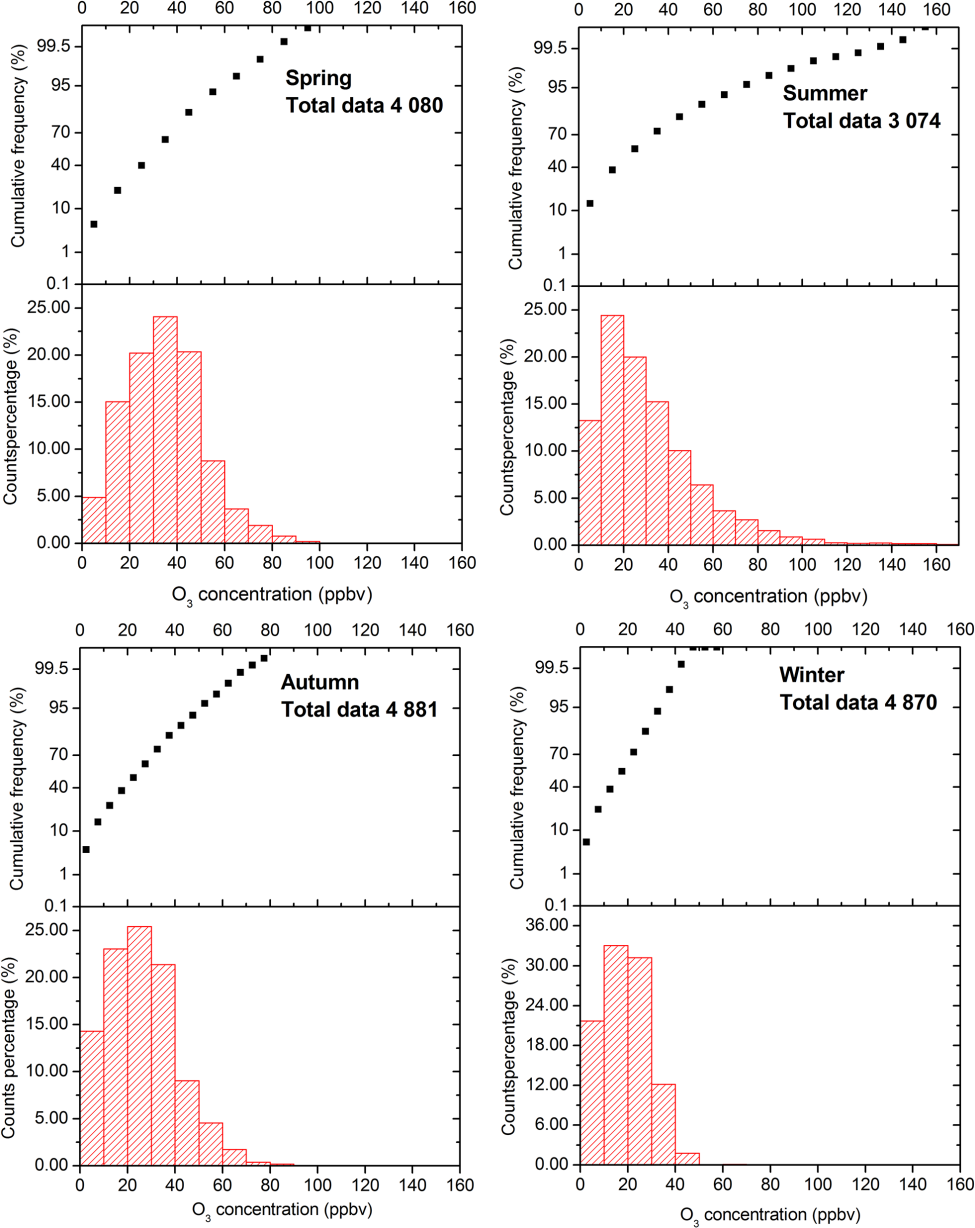
Frequency distribution of 1-h O_3_ data for different seasons (Spring: March, April and May, Summer: June, July and August, Autumn: September, October and November, Winter: December, January and February).

In Fig 6, the counts percentages and cumulative frequency distributions of hourly average O_3_ concentration in four seasons are shown. The counts of available hourly samples from spring to winter were 4080, 3074, 4881, and 4870, respectively. In the winter season, nearly 54.7% of the hourly readings were less than 20 ppbv, and only 6 hourly samples exceeded 50 ppbv (maximum 70.7 ppbv). However, during summer, only 37.7% of the hourly values were lower than 20 ppbv and the maximum concentration was extremely higher than those in other seasons. The seasonal averages in spring, summer, autumn and winter was 34.6±11.7, 31.9±17.1, 26.3±10.2 and 19.1±7.1 ppbv, respectively, which re-confirmed the fact that O_3_ levels were higher in spring and summer while lower in winter at Shanghai.

**Fig 7:**
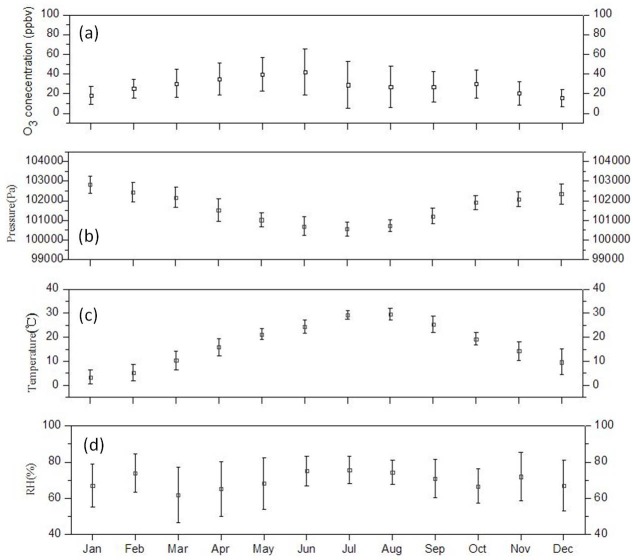
Monthly average of (a) O_3_ concentrations, (b) pressure, (c) temperature and (d) relative humidity from March 2010 to March 2013.

Fig 7 presents the monthly variations in O_3_ concentration with respect to atmospheric pressure, ambient temperature, and relative humidity. Fig 7(A) shows typical seasonal cycles, with maximum O_3_ in spring and summer whereas minimum in winter. The monthly average O_3_ in June (41.1 ppbv) was nearly three times as high as in December (15.2 ppbv). The average concentration gradually increased from 18.1 ppbv in January to the annual peak around 40 ppbv in May and June, and then quickly declined to around 30 ppbv in July to October, afterwards it began to decrease until December. The peaks observed in late spring and early summer have been widely reported in eastern China [7, 32, 33].

Temperature is a critical factor in determining the rates of chemical reactions, and also is dominating to the variations of ozone concentration [34]. In our observations, O_3_ concentration increased with ascending temperature until June, and then decreased with descending temperature from September to December. As expected, the atmospheric conditions in summer gradually became more favorable for photochemical formation of ozone. The positive correlation between monthly averaged temperature and ozone was found during the increase and decrease process of O_3_ concentration, respectively, which agreed with the general understanding of ozone chemistry and dynamics [34, 35]. Previous studies have observed that hot and dry environment favors the production of ozone [36]. However, in the present study, the highest monthly temperature occurred in August, whereas the highest monthly O_3_ concentration was in June. The decline in O_3_ level from June to September might be influenced by the Asian summer monsoon, which brings oceanic air containing less ozone to the region [12, 32, 37]. Previous studies suggested that ozone concentration is negatively correlated with the relative humidity due to the reaction with water vapor, which was not found during this long term observation, as shown in Fig 7(D) [38].

**Fig 8:**
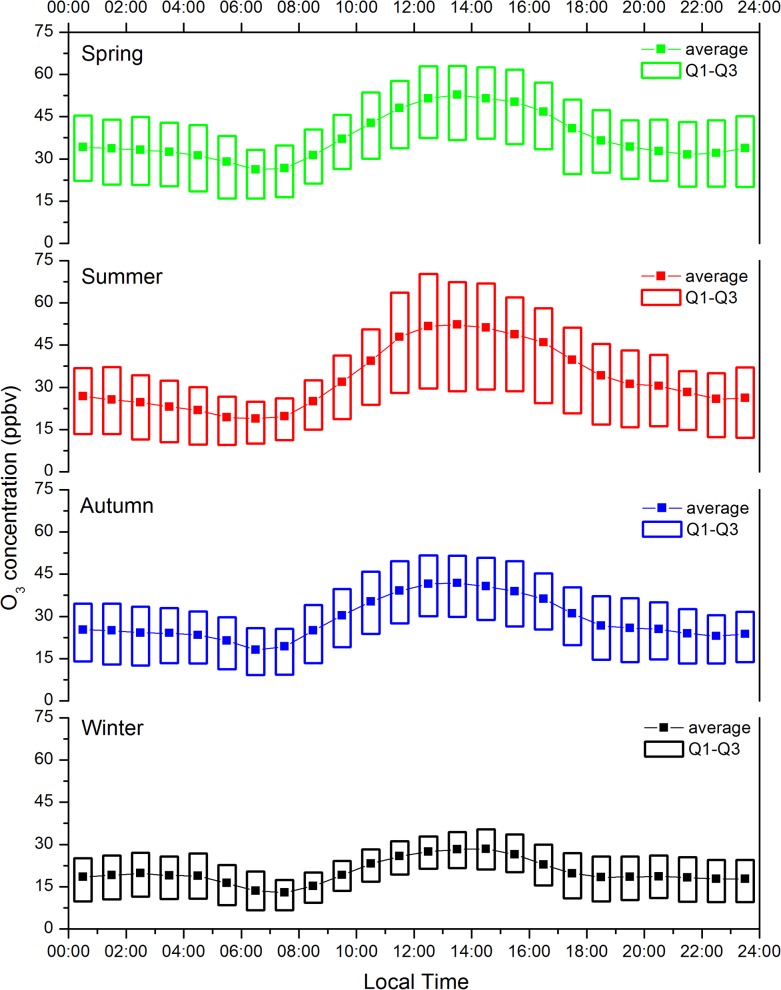
Diurnal cycle of averaged O_3_ concentrations for different seasons.

Fig 8 shows the diurnal variations in hourly O_3_ concentrations for different seasons between March 2010 and March 2013. Similar diurnal patterns were discovered among different seasons that higher O_3_ levels appeared around 12:00~14:00, whereas lower levels occurred in the early morning from 06:00 to 08:00. O_3_ concentration decreased slowly from 00:00 to 04:00 then declined significantly during early morning from 05:00 to 07:00, reaching the daily minimum. After sunrise, ozone concentration increased rapidly to its peak in the early afternoon, then decreased sharply in late afternoon. Additionally, the O_3_ diurnal cycle showed much larger amplitudes in warmer seasons than during cold seasons.

### Relationships between O_3_, NO_2_ and HONO

O_3_ is formed photochemically from the photolysis of NO_2_, and O_3_ reacts rapidly with NO reactions to produce NO_2_. As a result, NO, NO_2_, and O_3_ are in photoequilibrium, with no net formation or loss of O_3_. However, in the presence of VOCs, OH radicals react with VOCs to form intermediate RO_2_ radicals (R3). These RO_2_ radicals react with NO, which facilitate the cycling of NO to NO_2_ and O_3_ formation [39]. The photolysis of HONO (R2) after sunrise leads to the productive OH radicals during early morning, which may result in the net formation of O_3_.

HONO+hν→OH+NO(300nm<λ<405nm) (2)

$$OH\;+VOC\;\left(+\;O_{2}\right)\to \;RO_{2}$$ (3)

$$RO_{2}+NO\to RO+NO_{2}$$ (4)

**Fig 9:**
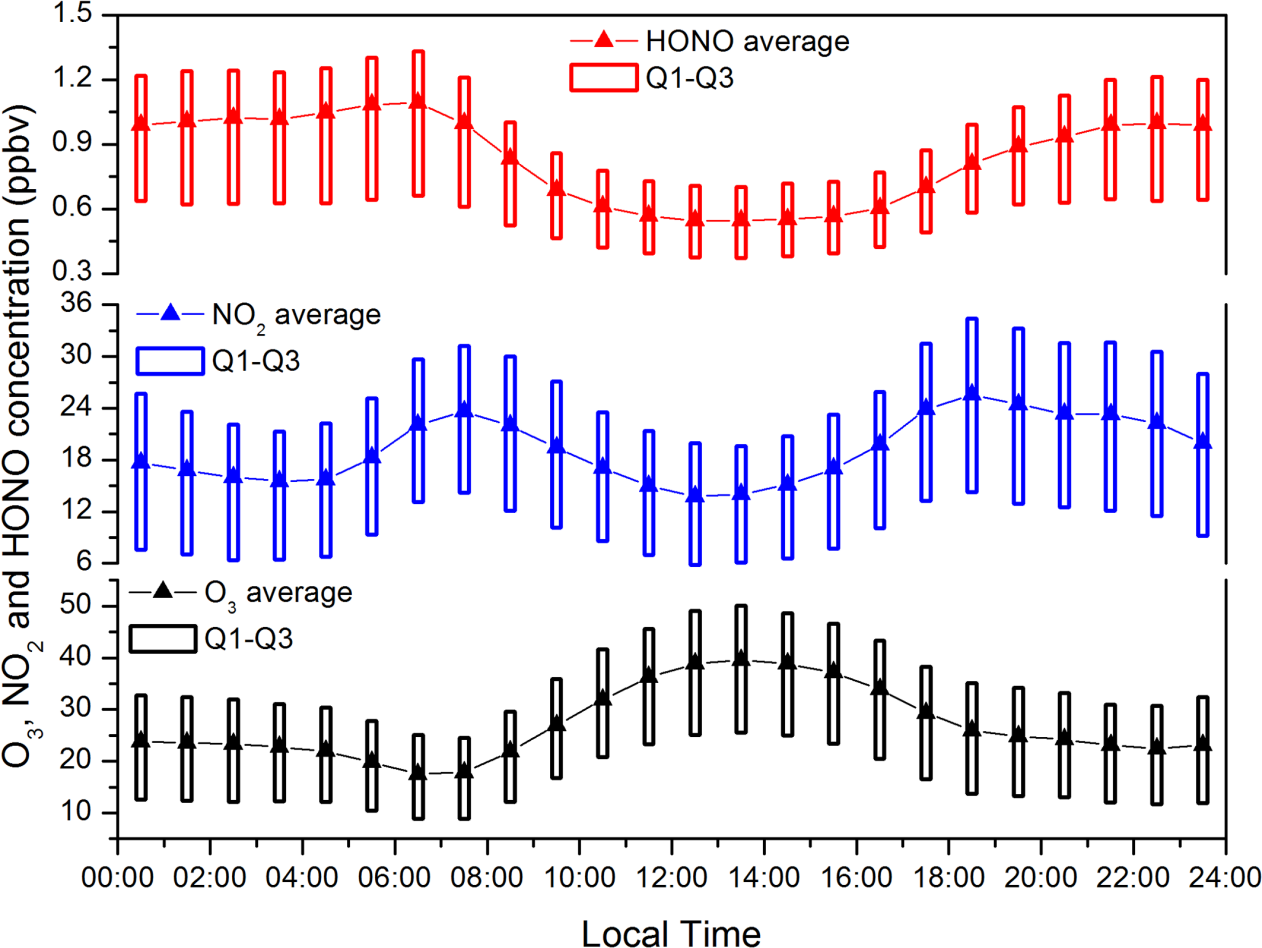
Diurnal cycle of averaged O_3_, NO_2_, and HONO concentrations during March 2010 to March 2013.

Fig 9 shows the diurnal cycle of NO_2_, HONO, and O_3_ concentrations from March 2010 to March 2013. NO_2_ concentration was among the highest around 07:00 and 18:00, which can be explained by the increased vehicular emissions during the daily peak traffic periods. From 07:00 onwards, NO_2_ was converted to NO and O_3_ through photolysis, while NO was converted back to NO_2_ reaction with O_3_, which also led to O_3_ consumption [40]. Unfortunately, data on NO were unavailable in this study, but it can still be speculated that NO concentration may increase with NO_2_ during the early morning, owing to the increased emissions from vehicles. Thus, O_3_ was mainly consumed by reaction with NO in the early morning. Additionally, weak UV intensity during the morning slowed the rate of NO_2_ conversion to O_3_, leading to the low concentration of O_3_ observed around 06:00. The same reasons resulted in the rapid decrease of O_3_ in late afternoon. Induced by solar radiation, O_3_ concentration began to increase gradually after sunrise, while NO_2_ concentration continued decreasing until reaching its lowest level around 12:00. HONO concentrations dropped sharply from 6:00 to 11:00, remained low until 15:00 and then increased rapidly and remained high at night. The comparable concentration and similar diurnal pattern of HONO were also exhibited in another eastern Chinese urban site [41]. OH radicals produced by HONO photolysis could yield RO_2_ radicals via the reactions with VOCs. When RO_2_ radicals continuously converted NO to NO_2_, O_3_ reached the highest concentration in the daytime.

### High ozone days

As the DOAS system failed to work in May, June, July, and August 2011, and in June, and July 2012, the present section focuses on analyzing the data in 2010. High ozone days (HODs) were defined as days during which hourly average concentration exceeding Grade II of CAAQS of O_3_ (102 ppbv) appeared. Table 2 summarizes the basic condition of HODs, comprising 56 hourly O_3_ concentrations exceeding the threshold on 14 different days.

**Table 2:** Summary of high ozone days in 2010.

Year	2010			
Month	May	June	July	August
The number of HODs	1	6	3	4
The number of exceeding hours	5	26	10	15
Max. ozone (ppbv)	123.7	161.3	161.3	167.3

**Fig 10:**
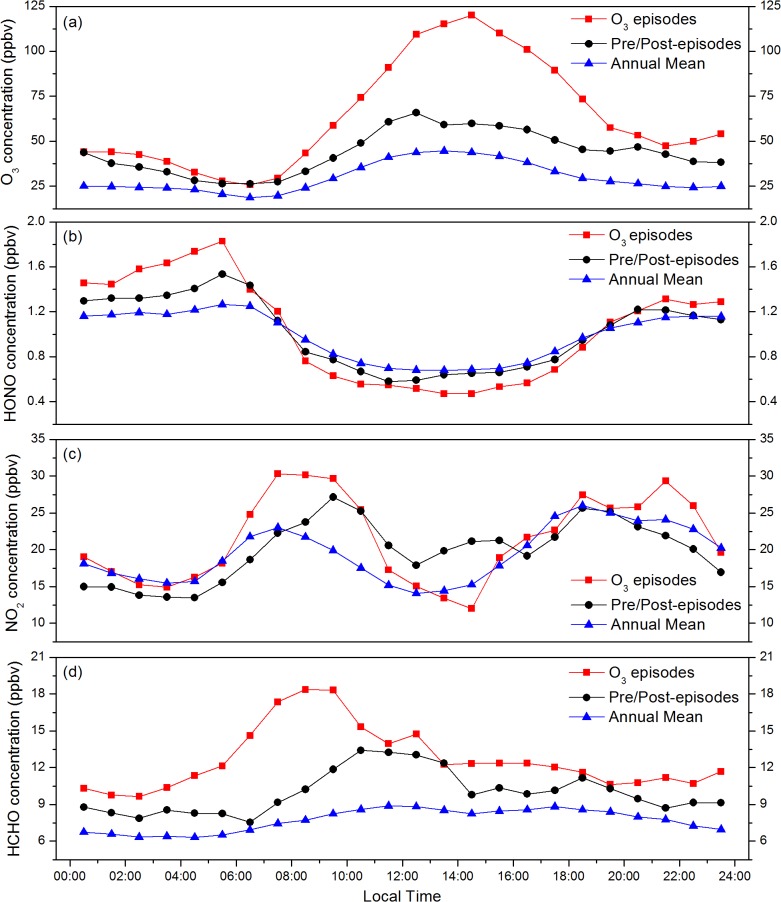
Diurnal variations of (a) O_3_, (b) HONO, and (c) NO_2_ averaged for HODs, pre-/post-episodes and annual mean in 2010.

To determine the causes of high O_3_ episodes, we depicted the diurnal concentrations of O_3_, HONO, NO_2_ and HCHO on the 14 selected HODs, together with the preceding and succeeding days for each, as well as the annual means (see Fig 10). Fig 10(A) shows a typical O_3_ diurnal cycle, with daily minimum in the early morning and maximum in the early afternoon. During the episode days, ozone concentration reached 120.1 ppbv at 14:00 (about 60 and 80 ppbv higher than those during pre-/post-episode and annual mean), suggesting the occurrence of intensive photochemical reactions. HONO concentrations between 22:00 and next 06:00 during O_3_ episodes were significantly higher than those during non-episode days, whereas the concentrations at noon were lower, which implies that more HONO was decomposed in daytime during O_3_ episodes (Fig 10(B)). Li [42] concluded that the addition of HONO sources significantly affects HOx (HOx = OH + HO_2_) in Mexico City, leading to a midday average increase in O_3_ of about 6 ppb. Czader [43] found that because HONO immediately photo-dissociates during daytime, its ambient mixing ratios were only marginally altered (up to 0.5 ppbv), but increases in hydroxyl radical (OH) and ozone concentration were obtained. In Shanghai, heterogeneous reactions has been considered to be the significant contributor to HONO formation [11, 25, 44], which may further impact the ozone formation. The sensitivity simulation without heterogeneous HONO sources indicated that the heterogeneous HONO formation would enhance daytime average O_3_ production by rate by ~6.8 pph h^-1^ on average in Shanghai due to the released OH via HONO photolysis [45]. Therefore, it can be inferred that HONO at high concentration during episode days was capable of increasing the O_3_ concentration. For NO_2_, the diurnal cycles show a morning/evening peak on episode and non-episode days alike (Fig 10(C)). The photolysis of NO_2_ from the morning peak resulted in O_3_ increase in daytime.

Comparison of the diurnal patterns for episode and non-episode days showed that levels of O_3_, HONO, and NO_2_ were higher during episodes. During the morning of HODs, increased solar radiation accelerated the rates of photolysis of NO_2_ and produced more O_3_, and HONO at high concentration was capable of producing abundant OH radicals via photolysis (R2). Then, OH radicals reacted with VOC to form RO_2_ radicals (R3), which subsequently reacted with NO to produce NO_2_ (R4). Increasing NO_2_ would prohibit the reaction of NO with O_3_ and reduce the consumption of O_3_. Furthermore, the photolysis of NO_2_ could enhance the increase of O_3_. As a result of the above steps, O_3_ was formed continuously and maintained high concentration.

### HYSPLIT back-trajectories for high ozone days

**Fig 11:**
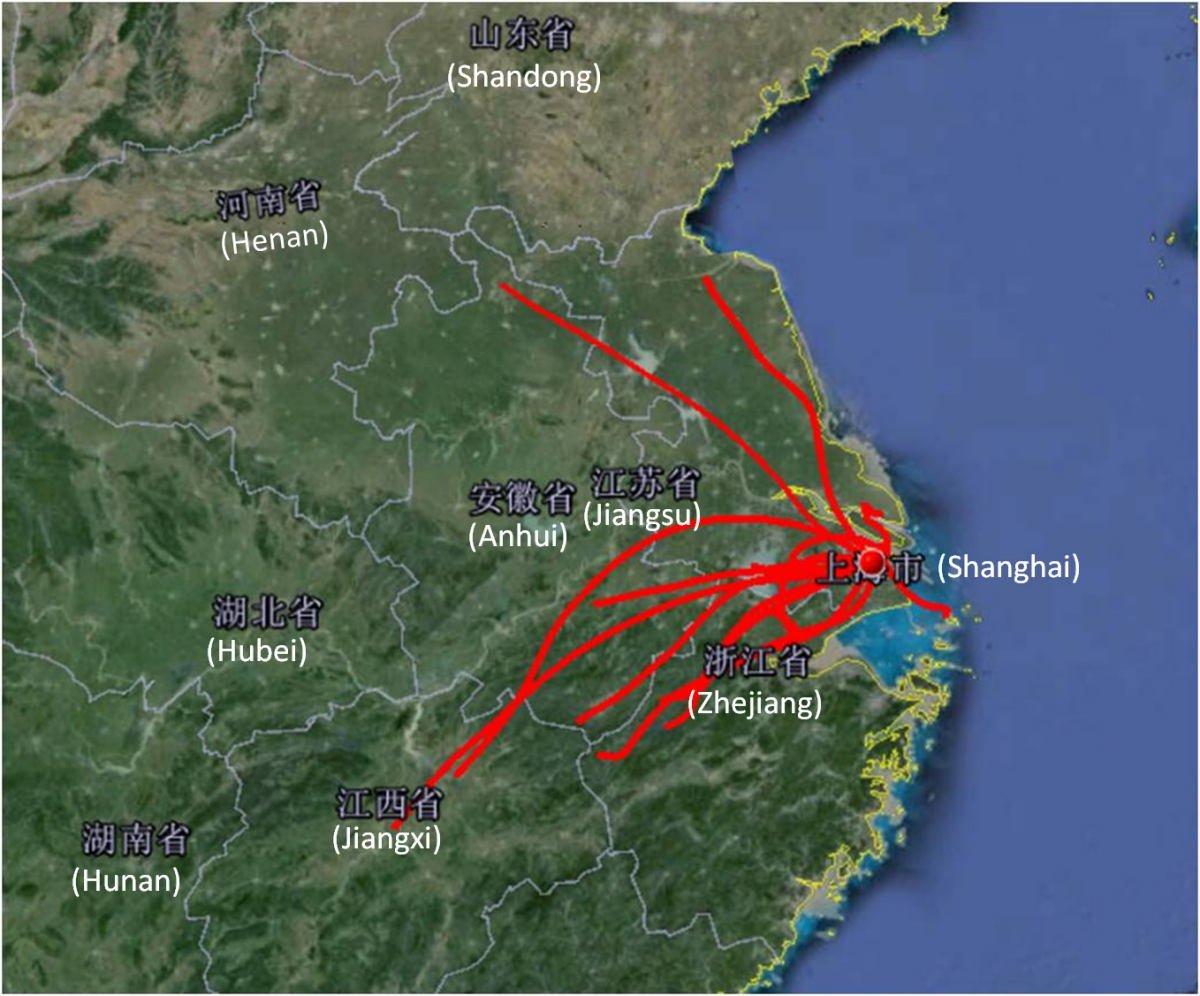
24-h air mass back-trajectory during HODs.

To assess the types of air mass transport processes during these HODs, 24-h backward trajectories were analyzed via running the HYSPLIT model for HODs once a day at 12:00 Chinese Standard Time (CST) at an altitude of 500 m above ground level, presented in Fig 11.

Table 3 shows the province and direction, from which the air masses originated, along with the transport distance. On 9 of the 14 HODs, air masses originated from the southwest inland, at distances ranging from 195 km (from Jiangsu Province on June 23) to 646 km (from Jiangxi Province on June 30). Two air masses coming from the northwest of Shanghai originated in Anhui and Jiangsu. Only one air mass originated over the sea, beginning 145 km away and arriving in Shanghai on June 15. Southwest of the FDU campus is the Jinshan chemical industrial zone, from where numerous chemical facilities emit considerable quantities of VOCs.

**Table 3:** Location, direction and distance of air masses trajectories in HODs.

Date	Location	Direction	Distance (km)
5.19	Anhui	Northwest	573
6.12	Jiangsu	Northwest	363
6.15	sea	Southeast	145
6.18	Zhejiang	Southwest	315
6.19	Zhejiang	Southwest	382
6.23	Jiangsu	Southwest	194
6.30	Jiangxi	Southwest	646
7.7	Jiangsu	North	112
7.29	Anhui	West	317
7.30	Jiangxi	Southwest	308
8.2	Jiangxi	Southwest	607
8.3	Zhejiang	Southwest	218
8.12	Zhejiang	Southwest	195
8.13	Anhui	Southwest	395

Based on the discussion about backward trajectories for the O_3_ periods, it is very likely that pollutants such as non-methane organic compounds (NMOC) were transported from petrochemical facilities located in Jinshan industrial zone. High concentrations of VOCs and ozone in transported air masses may be the cause of O_3_ increase during high ozone episodes. Since HCHO was regarded as an important indicator of NMOC emissions, it can be inferred from Fig 10(D) that air mass originated from Jinshan area may contain more VOCs and facilitate the O_3_ formation at downwind urban area during the O_3_ episodes. Moreover, the oxygenated VOCs play important roles in O_3_ formation were also demonstrated by some previous modeling and measurement studies in Shanghai [46].

### Case study for high ozone days

To further understand the factors leading to extremely high ozone levels, one episode was chosen for an in-depth analysis.

**Fig 12:**
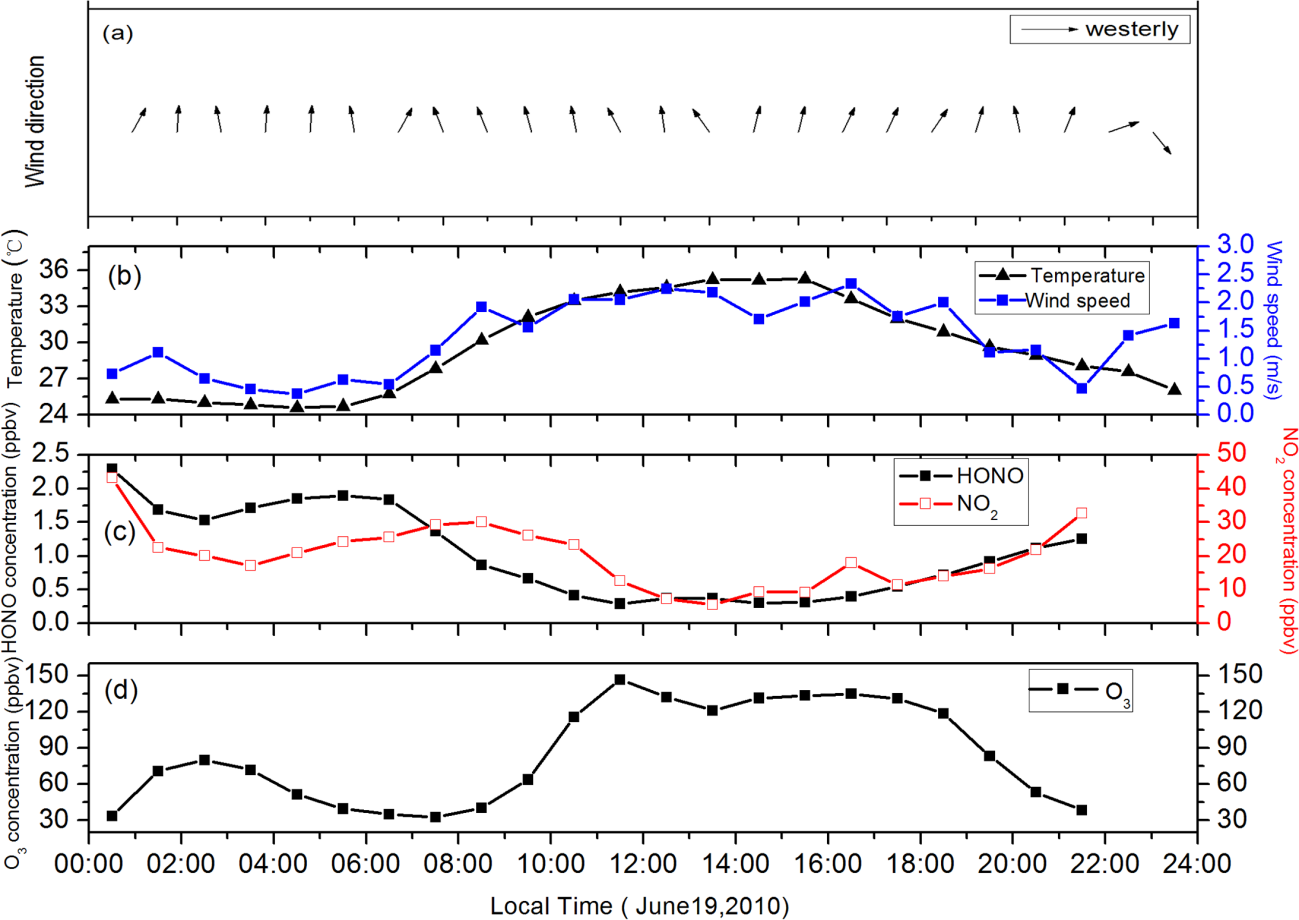
Wind direction (a), diurnal variations of (b) temperature and wind speed, (c) HONO and NO_2_, (d) O_3_ for case study.

**Fig 13:**
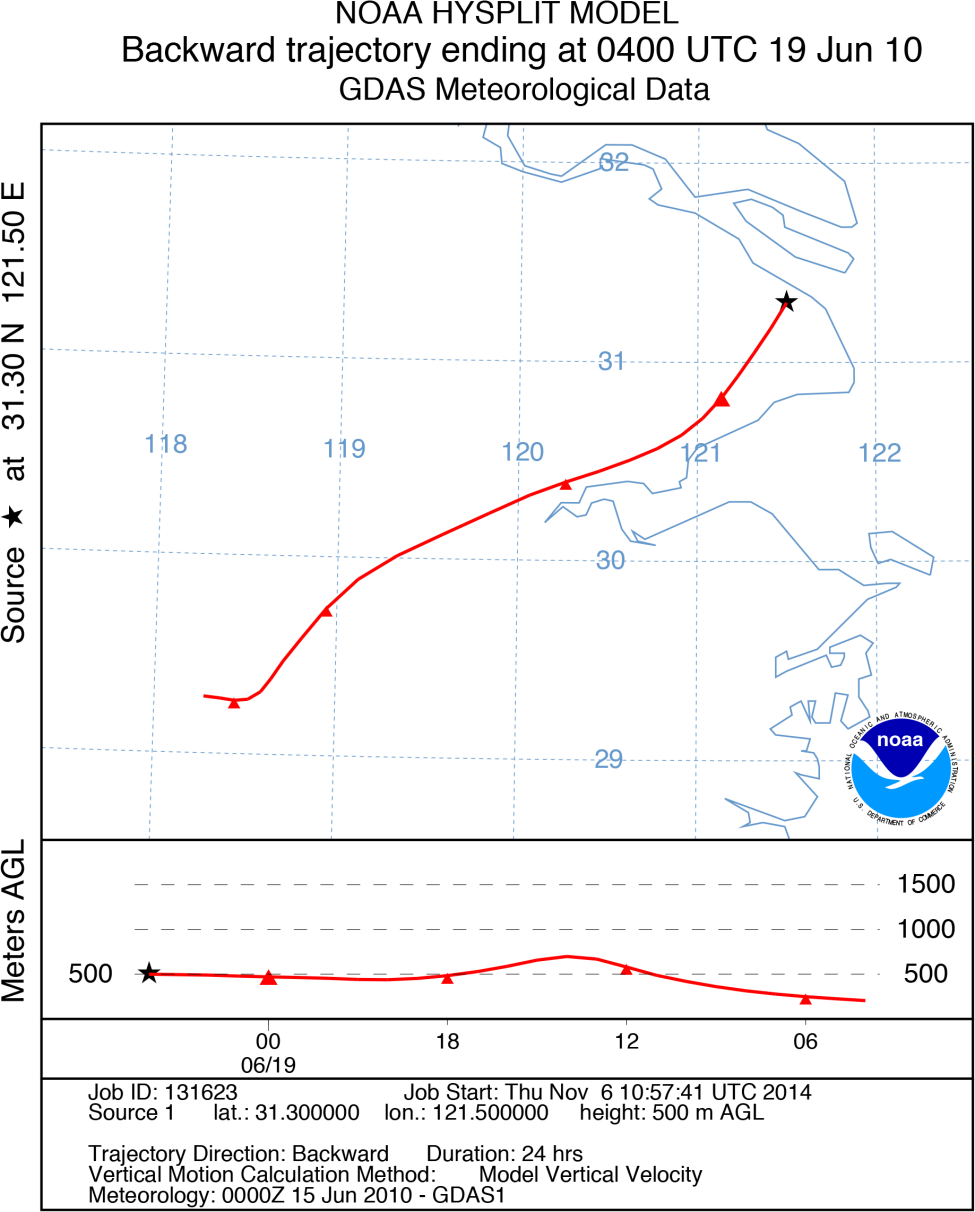
The 24-h air mass back-trajectory of ozone episode at 12:00 (CST) on June 19, 2010.

Fig 12(D) presents a high ozone episode on 19 June, 2010 that lasted for 9 h, from 10:00 to 20:00. HONO and NO_2_ also reached high levels on 19 June. HONO concentration peaked at 2.2 ppbv at midnight and remained approximately above 1.8 ppbv from 04:00 to 07:00 (Fig 12(C)) before high O_3_ occurred. When O_3_ concentration began to increase, that of NO_2_ and HONO began to decrease and then remained at low concentrations. Fig 12(B) shows diurnal temperature and wind speed. The highest temperature in this case was up to 36°C. The photolysis rates of NO_2_ were rapidly enhanced under strong solar radiation. NO_2_ level remained relatively low at noon and produced more O_3_ during this cloudless day. Additionally, the wind speed was less than 2.5 m/s, which inhibited the diffusion of O_3_. HYSPLIT 24-h backward trajectory analysis is presented in Fig 13. There is an obvious transport of air mass from Zhejiang at a height of 500 m. The air mass passed through Jinshan area before arriving the measurement site. As shown in Fig 12(A), the main wind direction was southwest, which are consistent with the result of backward trajectory and further suggests that air mass from the Jinshan chemical region influenced urban O_3_ concentration. The trajectory remained in the lower atmosphere until arriving at Shanghai, indicating an extensive region of stable meteorological conditions.

The photolysis of HONO was estimated from June 19 to 21, 2010 by the Tropospheric Ultraviolet and Visible (TUV) Radiation Model (http://cprm.acd.ucar.edu/Models/TUV/). The production of OH radical from HONO photolysis was obtained from the modeled photolysis frequencies, J(HONO), and the mixing ratios of HONO by R5 [47].

$$P_{OH}=J\left(HONO\right)\;\times \left[HONO\right]$$ (5)

**Fig 14:**
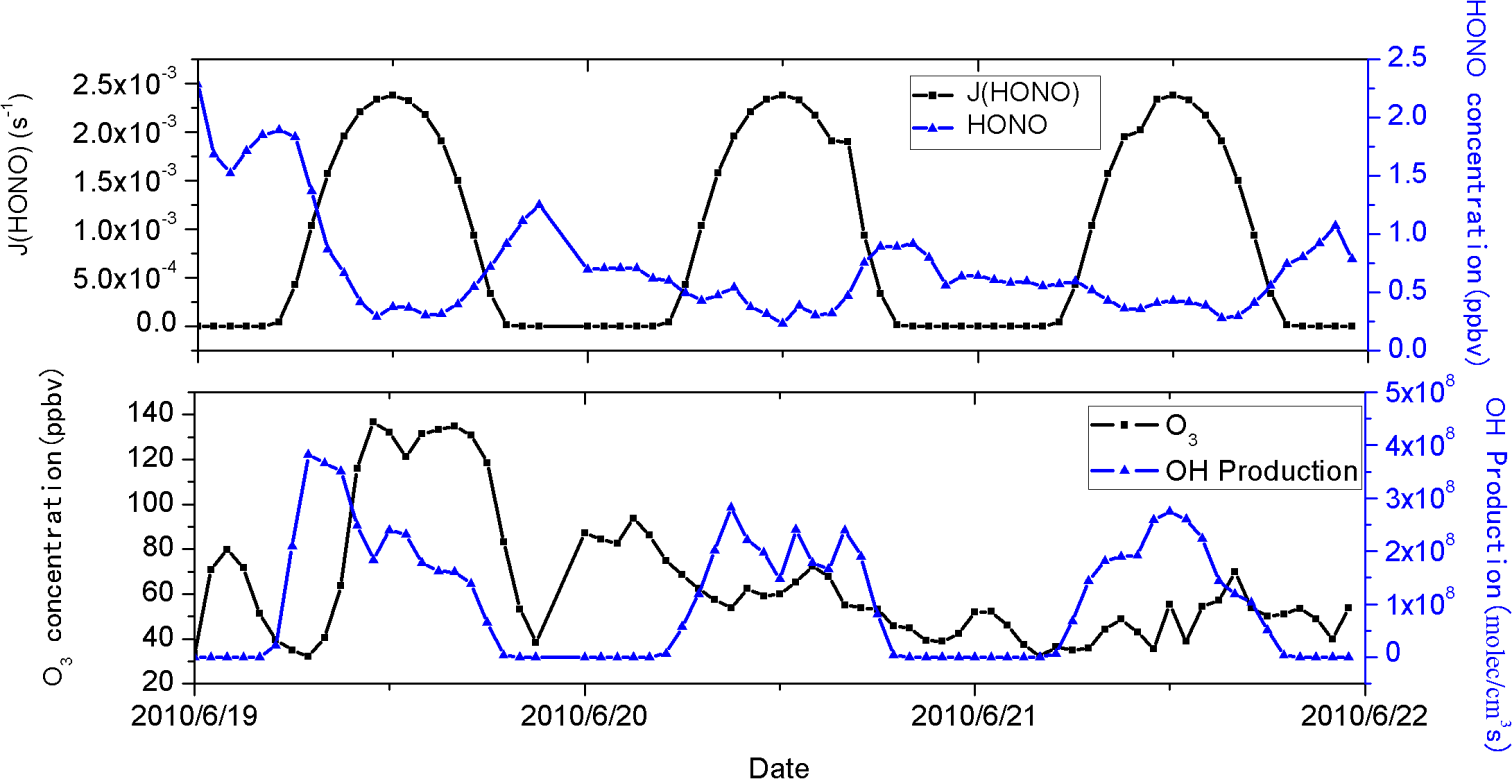
Rate of HONO photolysis and OH radical production, and concentrations of O_3_ and HONO from June 19 to June 21, 2010.

Fig 14 shows the rates of HONO photolysis and OH radical production, as well as concentrations of O_3_ and HONO for June 19–21, 2010. The concentrations of O_3_ and HONO on June 19 were much higher than on June 20 and 21. The production of OH radicals on June 19 (the maximum 3.8×10^8^ molec cm^-3^ s^-1^) was significantly greater than June 20 (the maximum 2.8×10^8^ molec cm^-3^ s^-1^) and June 21 (the maximum 2.7×10^8^ molec cm^-3^ s^-1^). High concentrations of HONO produced more OH radicals, which have a greater impact on ozone concentrations.

In this case, the sunny and hot conditions facilitated NO_2_ photolysis to form O_3_; the low wind speed inhibited the diffusion of O_3_; the transfer of air mass through the Jinshan chemical industrial zone influenced urban O_3_ concentration; the additional OH radicals resulting from high concentration of HONO were capable of increasing the O_3_ concentration.

## Conclusions

Utilizing the DOAS technique, a long-term measurement of ground-level ozone was originally performed from 2010 to 2013 over Shanghai, China. Good correlation with SEMC data suggests that the results measured by DOAS method are reliable (R = 0.92 for O_3_ and R = 0.84 for NO_2_). In our study, 56 hourly concentrations (on 14 separate days) in 2010 were found to exceed the Grade II limit of 200 μg/m^3^ specified for 1-hour ozone concentration. Generally, the 1-hour average concentrations of O_3_ were 27.2 ± 17.0 ppbv. Considering seasonal variability, O_3_ levels in late spring and early summer were the highest, and the lowest in winter. The highest monthly average O_3_ concentration in June (41.1 ppbv) was nearly three times as high as the lowest level recorded in December (15.2 ppbv). Seasonal and diurnal patterns of surface ozone were consistent with previous studies and were intimately associated with meteorological factors.

HODs were analyzed to establish the formation mechanism of high-ozone episodes, revealing higher levels of precursory species for O_3_, including HONO and NO_2_, on ozone-polluted days than on non-episode days. Photolysis of HONO can generate OH radicals, which further react with VOCs to produce RO_2_ radical. New formation of radicals consumed NO and produced NO_2_, resulting in further generation of ozone. Twenty-four-hour back-trajectory analysis showed that most of the air masses during HODs passed through the area occupied by the Jinshan chemical industry, which is a source of considerable VOC emissions. The provision of volatile organic compounds in Jinshan facilitates reaction with OH radicals, converting NO to NO_2_, resulting in increased ozone concentration.

The case study also illustrated the obvious influence of meteorological factors on ozone concentration. The occurrence of high O_3_ concentrations during daytime was attributed to the relatively high temperature, which favor the photochemical reactions to produce O_3_. Furthermore, the stable meteorological conditions, characterized by low wind-speed and compressed boundary layer, inhibited ozone dispersion. Air masses during high O_3_ episodes were transported through the chemical industrial region of Jinshan to the measurement site. The TUV Radiation Model suggests that high concentrations of HONO produced more OH radicals, which greatly increase ozone concentrations, in estimation of the photolysis rate of HONO.
